# Poultry fecal imagery dataset for health status prediction: A case of South-West Nigeria

**DOI:** 10.1016/j.dib.2023.109517

**Published:** 2023-08-23

**Authors:** Halleluyah O. Aworinde, Segun Adebayo, Akinwale O. Akinwunmi, Olufemi M. Alabi, Adebamiji Ayandiji, Aderonke B. Sakpere, Abel K. Oyebamiji, Oke Olaide, Ezenma Kizito, Abayomi J. Olawuyi

**Affiliations:** a College of Computing and Communication Studies, Bowen University, Iwo, Nigeria; b College of Agriculture, Engineering and Science, Bowen University, Iwo, Nigeria; c Computer Science Department, University of Ibadan, Ibadan, Nigeria

**Keywords:** Chicken Poo, Dataset, Machine learning, Image processing, Deep learning technique

## Abstract

Feces is one quick way to determine the health status of the birds and farmers rely on years of experience as well as professionals to identify and diagnose poultry diseases. Most often, farmers lose their flocks as a result of delayed diagnosis or a lack of trustworthy experts. Prevalent diseases affecting poultry birds may be quickly noticed from image of poultry bird's droppings using artificial intelligence based on computer vision and image analysis. This paper provides description of a dataset of both healthy and unhealthy poultry fecal imagery captured from selected poultry farms in south-west of Nigeria using smartphone camera. The dataset was collected at different times of the day to account for variability in light intensity and can be applied in machine learning models development for abnormality detection in poultry farms. The dataset collected is 19,155 images; however, after preprocessing which encompasses cleaning, segmentation and removal of duplicates, the data strength is 14,618 labeled images. Each image is 100 by 100 pixels size in jpeg format. Additionally, computer vision applications like picture segmentation, object detection, and classification can be supported by the dataset. This dataset's creation is intended to aid in the creation of comprehensive tools that will aid farmers and agricultural extension agents in managing poultry farms in an effort to minimize loss and, as a result, optimize profit as well as the sustainability of protein sources.

Specifications Table

**Table:** 

Subject	Applied Machine Learning
Specific Subject Area	Computer Vision Technique for poultry farm health status prediction
Type of Data	Image
How data were acquired	Image data was gathered with mobile smartphone connected to the Edge Impulse Platform and equipped with a 13-megapixel camera. Data was collected for a period of three-month: February to April 2023, from poultry farms on Bowen University campus and poultry farm around Iwo community. Animal research scientists and poultry farmers were involved in the labelling and annotation of the images.
Data format	Raw Image
Description of Data Collection	A total of 14,618 images were annotated with 100 by 100 pixel jpeg size. The data were grouped into 2 distinct folders labeled as “Healthy” or “Unhealthy.”
Data source Location	• Institution: Bowen University, Poultry Research Farm, Bowen University Commercial Farm • City/Town/Region: Iwo • Country: Nigeria
Data Accessibility	Repository name: Mendeley Data Data identification number: doi: 10.17632/8pnbzpt2k9.1 Direct URL to data: https://data.mendeley.com/datasets/8pnbzpt2k9/1 https://zenodo.org/record/8122337

## 1 Value of the Data

• Image dataset of the chicken fecal have potential value in early detection mechanisms and diagnosis of poultry diseases [1].
• There is paucity of publicly available datasets of poultry fecal images for poultry diseases diagnosis.
• The dataset is applicable in the development of computer vision based models for object detection, image segmentation and classification.
• The dataset will give more insights beyond human physical observation for accurate and precise detection of poultry diseases. It will aid agricultural extension agents in engaging poultry farmers in a more effective training and orientation.

## 2 Objective

This dataset was created with the intention of providing an open, usable, and high-quality machine learning dataset for the detection of anomalies in poultry farms. Localized and well-labeled datasets are necessary for the creation of useful and efficient machine learning systems for use in the real world. Image dataset offers a method for quickly identifying anomalies, which will aid in addressing the problem of economic loss brought on by the high rate of poultry birds casualties in Nigeria [1]. A variety of machine learning use-cases, including image segmentation, detection and classification can be delivered by the dataset's annotated formats [2]. Other researchers can use the dataset to model the development of poultry and chicken diseases, which can help with speedy response and lessen the issue of food security in Africa [3,4].

## 3 Data Description

The paper shows image dataset of chicken fecal, collected from poultry farms located in Bowen campus and Iwo community. The poultry farms rear two different species of poultry birds: broiler and layer. There are 14,618 annotated photos in the collection, each measuring 100 by 100 pixels size and having joint photographic expert group (jpeg) format [5]. Data were submitted into two distinct folders in the repository—healthy and unhealthy—each of which contained four files of photographs taken at various times during the day—morning, afternoon, night—as well as additional images uploaded in a zip folder. The healthy folder contains 60 physical instances while the unhealthy folder has 34 physical instances. Each of this instance have an average variability of 10 accounting for light intensity, orientation, etc.

**Fig. 1:**
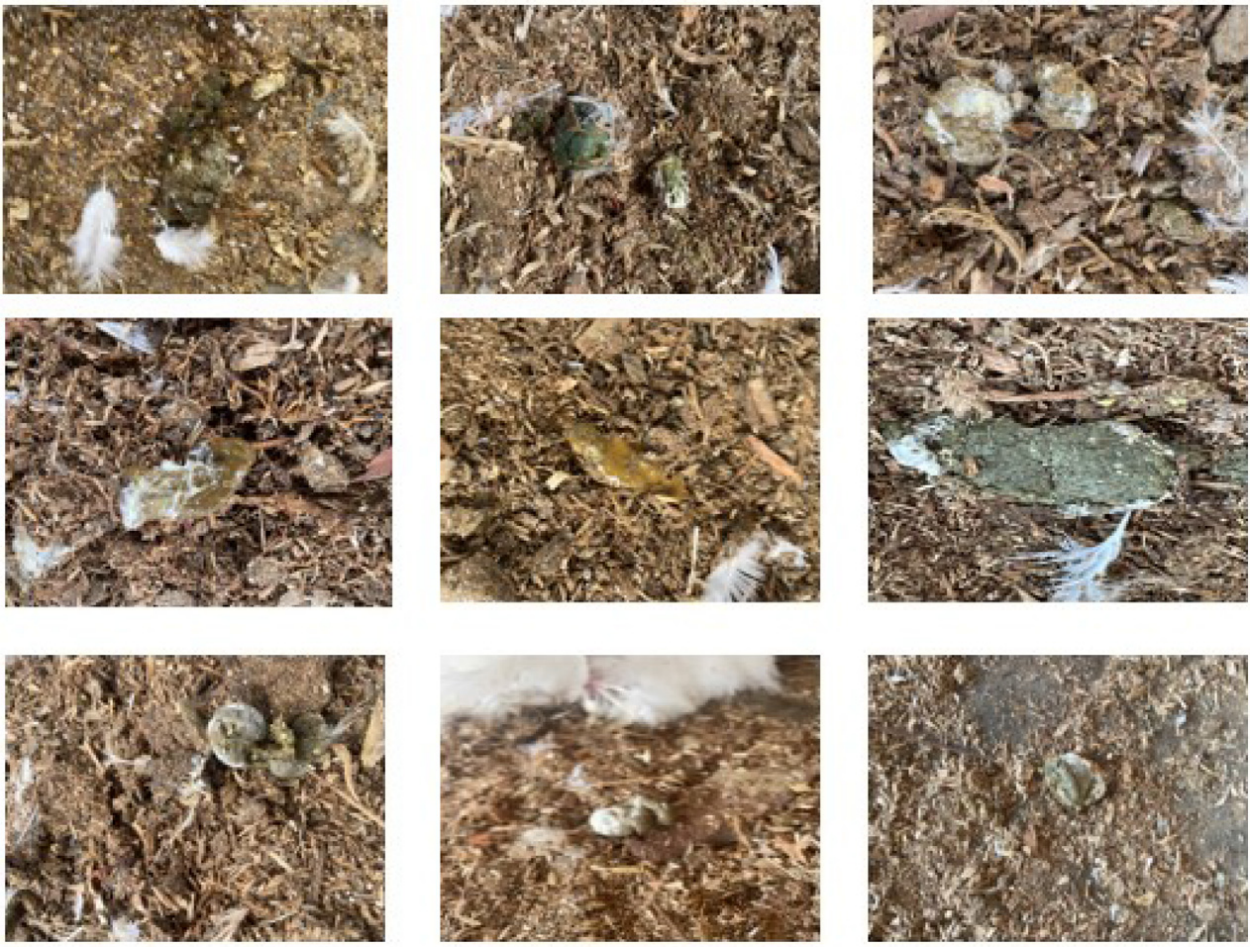
Samples of Fecal Images. (For interpretation of the references to color in this figure legend, the reader is referred to the web version of this article.)

To make data uploading and downloading simple, images were divided into separate folders [6]. Fig. 1 displays image samples of both healthy and unhealthy chicken feces.

## 4 Experimental Design, Materials and Methods

### 4.1 Field Data Collection

The data contains images of chicken fecal collected by the Computing & Analytic Research Group, Bowen University, Osun, Nigeria. Poultry farms on Bowen University campus and some selected farms in Iwo community were used for data gathering for three-month period, from February to April 2023. Burst mode on mobile device: Samsung Galaxy 01 equipped with 13 megapixels was used to capture image data. Burst mode in still-cameras involves capturing multiple images in quick succession. Burst mode is achieved by either pressing the shutter button or holding it down. Animal Scientists and poultry farmers were involved in data verification and annotation.

### 4.2 Data Preprocessing

Raw Images were preprocessed which include cleaning, renamed, resizing and annotation [4]. The dataset was labeled on Edge Impulse studio [7] using Yolo version 5 [8]. In 2015, Joseph Redmon, Santosh Divvala, Ross Girshick, and Ali Farhadi developed a cutting-edge, real-time object identification method that was pre-trained on the COCO dataset. To process a whole image, it only employs one neural network. The program divides the image into areas, and for each region, it forecasts probability and bounding boxes.

**Fig. 2:**
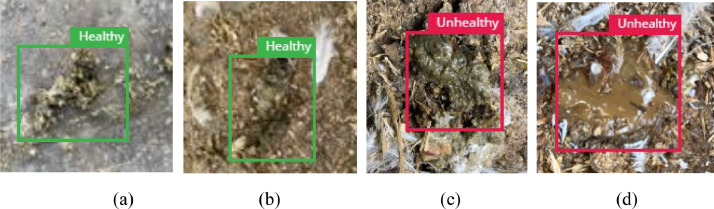
Sample Images of Fecal (a) Healthy (b) Healthy (c) Non Healthy (d) Non Healthy. (For interpretation of the references to color in this figure legend, the reader is referred to the web version of this article.)

The preprocessed data was then uploaded to the open database repository. Table 1 presents statistics of fecal images collected for different time of the day while Table 2 shows the statistics after preprocessing. The image labeler in MATLAB which is an open-source tool was used to annotate the images as shown in Fig. 2. The MATLAB image labeler provides an interactive approach to create region of interest (ROI) labels [9].

**Table 1:** Chicken fecal Image Dataset before Preprocessing.

Category	Morning	Afternoon	Night	Others	Total
Healthy	10878	1765	2871	767	16281
Non-healthy	5994	2032	4590	258	12874

**Table 2:** Chicken fecal image dataset after preprocessing.

Category	Morning	Afternoon	Night	Others	Total
Healthy	3588	1765	1871	767	7991
Non-healthy	2768	2032	1569	258	6627

## Ethics Statements

The animal study was reviewed and approved by Bowen University Research Ethical Board. The research was conducted under the required ethical guidelines issued and signed by the Director of Research and Strategic Partnership for the Board. Ethical Approval Number: BUREC/COCCS/CSC/0003.

## CRediT authorship contribution statement

**Halleluyah O. Aworinde:** Conceptualization, Methodology, Data curation, Writing – original draft, Visualization, Investigation, Writing – review & editing. **Segun Adebayo:** Conceptualization, Methodology, Data curation, Writing – original draft, Visualization, Investigation, Writing – review & editing. **Akinwale O. Akinwunmi:** Data curation, Visualization, Investigation. **Olufemi M. Alabi:** Data curation, Writing – review & editing. **Adebamiji Ayandiji:** Methodology, Data curation, Writing – original draft. **Aderonke B. Sakpere:** Methodology, Data curation, Writing – review & editing. **Abel K. Oyebamiji:** Data curation, Visualization, Investigation. **Oke Olaide:** Data curation, Visualization, Investigation. **Ezenma Kizito:** Data curation, Visualization, Investigation. **Abayomi J. Olawuyi:** Data curation, Visualization, Investigation.

## Data Availability

Poultry Birds Poo Imagery Dataset for Health Status Prediction: A Case of South-West Nigeria (Original data) (Mendeley Data).
